# Ischaemic Heart Disease: Accuracy of the Prehospital Diagnosis—A Retrospective Study

**DOI:** 10.1155/2013/754269

**Published:** 2013-03-28

**Authors:** Louise Houlberg Hansen, Søren Mikkelsen

**Affiliations:** ^1^Department of Anaesthesiology and Intensive Care, Odense University Hospital, Sdr. Boulevard 29, 5000 Odense C, Denmark; ^2^Mobile Emergency Care Unit, Department of Anaesthesiology and Intensive Care, Odense University Hospital, Sdr. Boulevard 29, 5000 Odense C, Denmark

## Abstract

*Purpose*. Correct prehospital diagnosis of ischaemic heart disease (IHD) may accelerate and improve the treatment. We sought to evaluate the accuracy of prehospital diagnoses of ischemic heart diseases assigned by physicians. *Methods*. The Mobile Emergency Care Unit (MECU) in Odense, Denmark, services a population of 260.000. All admissions in 2009 concerning patients diagnosed in the IHD category were assessed. Outcome and diagnosis of each patient were manually validated in accordance to the final diagnosis established following admission to hospital, using the discharge summary from the relevant department as reference. *Results*. 428 MECU runs with a prehospital diagnosis of IHD were registered. 422 of these were included in the study and 354 of those patients were suitable for this analysis. 73,4% of the patients hospitalized with a prehospital diagnosis of IHD were initially admitted to the relevant ward. Of these patients, 40,0% had their preliminary diagnosis of IHD confirmed. 14,1% of all patients admitted to the hospital were diagnosed with nonheart conditions. Preliminary diagnoses of STEMI had an accuracy of 87,5%. *Conclusions*. The preliminary IHD diagnoses assigned by the MECU physicians were acceptable. In case of STEMI patients the diagnostic accuracy was excellent. In this study there was an apparent overtriage.

## 1. Background

Ischaemic heart disease (IHD) as a part of a general cardiovascular disease is the leading cause of death in Denmark and the leading cause of admission to hospitals in Denmark [1]. Other industrial countries follow the same pattern [2].

Large randomised trials have demonstrated that fibrinolytic therapy can reduce mortality in patients with suspected acute myocardial infarction (AMI) [3]. Furthermore, it is known that reducing time to reperfusion decreases morbidity and mortality [3, 4]. This knowledge calls for making a fast assessment of the patient's risk of having acute coronary syndrome (ACS). The simple solution to this problem is to admit all patients, even the ones with low suspicion of acute ischaemia, to specialized cardiac centres.

This concept, “Chest Pain Clinics,” is increasingly gaining acceptance, but there are potential drawbacks to sending all patients with chest pain to the same department, regardless of eliciting factor. This practice may lead to a poor cost-effectiveness and overtriage.

To eliminate these drawbacks, a diagnostic tool is needed to separate the patients in need of fibrinolytic therapy from other patients. This is yet to be discovered and often the decision to admit a patient to a given department lies with the treating physician or other emergency staff when no physician is present.

Recent studies have assessed the accuracy of AMI or ST-elevation AMI (STEMI) diagnoses made by both paramedic and physicians in prehospital settings, thereby reducing time to reperfusion. The accuracy in these studies was at best acceptable [5–8].

A large study has evaluated the triage distribution in emergency departments in case of suspected ACS. In this study, only 23% of the patients, admitted with chest pains or other symptoms consistent with ACS, actually were experiencing ACS [9].

In most parts of Denmark, a physician along with an emergency medical technician is accompanying the ambulance in case of suspected severe emergencies, including heart diseases. We wanted to study the accuracy of the preliminary diagnoses made prehospitally in cases of suspected IHD.

## 2. The Mobile Emergency Care Unit

The Mobile Emergency Care Unit (MECU) in Odense, Denmark, operates as a part of a two-tiered system, in which the MECU is dispatched with an ordinary ambulance manned with two Emergency Medical Technicians (EMTs).

The MECU in Odense consists of one rapid-response car, operating all year round and manned with a specialist in anaesthesiology and an EMT.

The MECU covers an area of approximately 2.500 square km and services a population of 260.000.

The MECU is dispatched either by the dispatch centre on the basis of the information given by the caller, or by secondary request from the EMTs on the primary ambulance. One of the criteria for dispatching the MECU along with an ambulance is sudden loss of consciousness (see Table 1).

In a typical year, the MECU is handling 4900 calls (13.5 calls per day). Due to apparent overtriage at the dispatch centre, in 13% of the calls, the ambulance waives the MECU en route following initial contact.

As a result of coincident requests for assistance, 3,2% of the requests are left unanswered.

Following each MECU run, patient characteristics (including the patient's Civil Registration System number (or Social Security Number), forming a unique identification of the patient), tentative patient diagnosis, and the treatment administered are entered into the MECU database.

The aim of the study was to investigate the patients attended to by the MECU in Odense, Denmark, who were assigned the diagnosis ischemic heart disease in order to establish the accuracy of the diagnosis.

## 3. Methods

The study is a retrospective, descriptive study approved by the Danish Data Protection Agency (J.nr. 2010-41-5095).

All MECU runs in 2009 with a prehospital diagnose of IHD made by the MECU physician were included in this study. The 4 following diagnoses were included: DI 200: unstable angina pectoris, DI 209: stable angina pectoris, DI 219: myocardial infarction, and DZ 034: obs. AMI.

The MECU database contains information about the patients' main complaints and duration hereof, information about any previous IHD, the patient's vital parameters, assessment of the ECG, and the treatment administered.

Using the patients' personal identification numbers, we manually compared the MECU diagnoses to the final diagnoses assigned in the discharge summary from the relevant department at Odense University Hospital (OUH).

### 3.1. Statistics

Data are presented as absolute numbers, means, percentage, or medians with 25th and 75th percentiles as appropriate. Whenever possible, 95% confidence intervals were calculated.

## 4. Results

In 2009, 4952 MECU runs were completed. In 428 of these ambulance runs, the patients were assigned an IHD related diagnosis. Of these, 6 runs were excluded as the IHD diagnosis had been suggested by another physician before the arrival of the MECU physician. The 6 runs dispatched in order to administer analgesics to patients already in ambulances on their way to hospital.

Mean age, gender distribution, and previous history of IHD of the patients treated by the MECU are summarized in Table 2.

To compare the prehospital diagnoses with the final diagnoses in the discharge summary, the patients had to be admitted to OUH. Consequently, the 34 patients treated and then left at home plus the 34 patients admitted to other hospitals than OUH could not be a part of this analysis. Table 2 lists the final diagnoses of the patients admitted to OUH with prehospital diagnoses of IHD (expressed as *n*, % (and 95% CI)). 5 patients were lost to followup: 4 left the hospital without treatment and in one case, the patient's medical records could not be retrieved.

Other heart conditions were primarily atrial fibrillations and heart failures. The distribution of nonheart conditions is summarized in Table 3.

21 of the admitted patients (5,9%) were characterized by the MECU physician as “unlikely to have a heart condition” but were still admitted to hospital. Of those patients, one was subjected to intravenous rehydration to correct his state of dehydration, and the rest were discharged without any treatment and got no final diagnoses.

The MECU physician gave 48 patients prehospital diagnoses of AMI (both STEMI and non-STEMI) before admitting them to OUH. The distribution of their diagnoses at discharge is shown in Table 4.

32 patients in the AMI category received prehospital STEMI diagnoses. 28 of these had their STEMI diagnoses confirmed at hospital, 2 had transient ST-elevations on their initial ECG, but not STEMI, one had non-STEMI, and one had third degree AV block (the last mentioned was already noted by the MECU physician).

## 5. Discussion

The accuracy of the preliminary diagnoses made by the MECU physicians was acceptable. Of the triaged patients with any of the four prehospital diagnoses of IHD, 73,4% were later confirmed as having a cardiac disease and as such were initially admitted to the correct department. Of those patients, 40% had their prehospital diagnosis of IHD confirmed. Almost half of the patients (49,6%) triaged to the cardiologic department were observed for 2 days and then discharged without a confirmation of IHD.

In a study by Pope et al. only 23% of the patients presenting with signs of acute coronary ischaemia in emergency departments were assigned a final diagnosis of ACS. 94% of those were admitted to hospital, and 6% were sent home. Of the 77% without ACS, 59% were admitted to hospital. Ultimately, this gives a confirmation of the ACS diagnosis in 32,2% of the triaged patients [9]. In comparison, in our study we found that 29,4% of all patients had their prehospital diagnosis of IHD confirmed.

The attending physician assigns the prehospital diagnosis on the basis of the patients reported symptoms, the patients appearance and vital sign, and the prehospital recorded ECG. The prehospital ECG is transmitted to the receiving coronary department, enabling the prehospital physician to seek advice from a cardiologist. There are no clear guidelines for admitting patients with suspected IHD, so it is each physician's judgment, supported by the opinion of the cardiologist, when to admit a patient for further diagnostics and treatment.

Many studies have made assessments of the presenting symptoms in ACS. Some of them indicate that the presence or absence of specific symptoms makes a final diagnosis of ACS more or less likely [10, 11]. Other studies challenge the value of relying on symptoms alone in diagnosing ACS. One relatively recent paper concludes that many of the known “typical” symptoms of ACS, which are often considered to identify high-risk patients, may render no diagnostic value, while “atypical” symptoms actually make ACS more likely [12].

Consequently, the attending physician cannot rely on the patient's symptoms alone to make a decision of admitting the patient to hospital. This is in line with recommendations from the European Resuscitation Council regarding initial management of acute coronary syndromes [13]. Using only the ECG for triage decisions is also shown to be inadequate [2, 14], although obtaining an ECG demonstrating ischemia or injury leads the patient to be more than 2.5 times likely to have an ACS diagnosis than patients who prehospitally showed no ECG ischemia/injury [15].

In this study, we find that a large number of cases are overtriaged. This is in line with another Danish study in which more than half of the patients assigned the diagnosis IHD in the prehospital setting, in fact are diagnosed as having IHD [8].

Of the patients admitted to hospital, 11% are discharged immediately after being seen by a physician at the emergency room. The apparent overtriage might be the result of the MECU physicians' extra precaution. A longer time perspective and the hospital physician's opportunity to observe the effect of the instituted prehospital treatment could also be the reason for discharging some of the patients following the first examination at the hospital. The apparent overtriage may be influenced by the public's expectations toward the health care system. As earlier stated, 21 (5,9%) patients are admitted to hospital, even though the MECU physicians have considered them to be unlikely to suffer from a heart condition. In accordance with their discharge summary, none of these patients needed admission. A suggested solution to this kind of patients might be another prehospital diagnosis, for example, “unspecific chest pains,” which would indicate the MECU physicians doubt of heart related illness.

Another study from Germany implies that prehospital cardiologists admit fewer patients than prehospital anaesthesiologists because of their larger clinical experience, which in return make them more likely to underestimate the severity of the illness [14].

The art of correct triage is admitting as few patients as possible without compromising the low rate of patients left at home/sent home, which actually needed treatment for some kind of IHD. In this study we only assessed the triaged patients, so our rate of missed diagnoses is not a part of this analysis.

Symptoms similar to those accompanying ACS may indicate other life-threatening illness, for example, aortic dissection. No such severe cases were encountered in this study, but all of the patients with other heart conditions needed admission and they were all triaged to the correct department by the MECU physician.

As shown in Table 4, almost half of the patients (42%) with nonheart conditions have infections, mostly pneumonia. Temperature is not routinely monitored in patients suspected of suffering from IHD. Consequently, fever is often undetected in the prehospital settings, which complicate diagnosing an infection.

A recent review states that gastroesophageal disorders are the most common causes of noncardiac chest pains [16]. The discharge summaries of the patients with the final diagnosis “observation, IHD not confirmed” revealed that a considerable number of patients had symptoms hinting towards gastroesophageal reflux disease (GERD). This is not shown as a part of the results, because these findings are not stated as confirmed final diagnoses. Nevertheless, we must assume that a substantial number of patients with undetermined causes of chest pains may have GERD.

Most studies on prehospital diagnoses of IHD deal with the emergency staffs capability of recognising AMI or STEMI. In these studies, prehospital diagnosis of STEMI gives rise to prehospital reperfusion treatment and early reperfusion treatment with PCI [5–7]. This is also the case in Denmark; if the MECU physician makes a prehospital diagnosis of STEMI, the reperfusion team at the hospital OUH is alerted and the patient receives aspirin, clopidogrel or ticagrelor, and heparin before being transported directly to the reperfusion center, with the team waiting to preform percutaneous coronary intervention. This system calls for little margin of error in making the prehospital diagnosis. To make comparisons to other studies, the diagnoses, where the MECU physician was certain that the patient was experiencing an AMI, were assessed (Table 5). This implies that when the MECU physician is sure of an AMI diagnosis, it is correct in more than 2/3 of the cases (70,8%) and initially, almost everyone (97,9%) was admitted to the correct department.

Other studies on prehospital STEMI and AMI diagnoses have a true positive diagnosis value of 90%, 80%, and 95%, respectively [5–7]. This is comparable to our results.

There are several limitations to our study. It was not possible to obtain the discharge summary from other hospitals than Odense University Hospital, so the 34 patients, who were admitted to other hospitals, were excluded from this analysis.

The other hospitals in the region are not capable of performing PCI, so most likely no patients with strong indications of ACS were admitted there.

As earlier discussed, patients receiving final treatment in their homes obviating admission to hospital were not subjected to followup and as such were not included. Also, cardiac patients erroneously assigned noncardiac diagnoses and thus admitted to noncardiac departments could not be entered into this analysis.

As this study was based on a retrospective assessment of the diagnosis codes in both the MECU database and in the discharge summary, coding errors might have contributed to accurate results.

## 6. Conclusions

The preliminary diagnoses made by the MECU physicians were fairly accurate when looking at all IHD patients and in accordance with other studies. In case of STEMI patients the accuracy was excellent. In this study an apparent overtriage is present.

## Figures and Tables

**Table 1:** MECU Dispatch criteria in the observation period.

Life threatening conditions:	
Sudden loss of consciousness	
Absense of breathing	
Noisy or otherwise impaired breathing	
Possible life threatening conditions:	
Dyspnea	
Severe chest pain	
Sudden onset of serious headache	
Impaired breathing in infants and children	
Suspected serious illness in children or infants	
Sudden onset of severe oral or rectal bleeding	
Sudden onset of bleeding in pregnant women beyond 20th gestational week	
Accidents implying a risk of life threatening conditions:	
Motorway accidents	
On highways	
High velocity car crash	
Entrapment	
Roll-over	
Lorry or bus involved	
Motorcycle involved	
Pedestrian against car/motorcycle	
Other accidents	
Fall from heights	
Entrapped persons	
Accidents with bleeding victims	
Accidents involving horses	
Gunshot or stab wounds towards torso, neck, head	
Hanging	
Drowning	
Burns involving face or exceeding 20% (adults) or 10% (infants and children) of body surface	
Area	
Accidents involving trains or aeroplanes	
Fire implying a risk of damage to people	
Chemical exposure	

**Table 2:** Characteristics of the study group (*n* = 422).

	Median or *N* (%)
Age (years)	68,6
Known earlier IHD	260 (61,6)
Men	277 (65,6)
Women	145 (34,4)

**Table 3:** Final diagnoses of the patients admitted at OUH (*n* = 354).

	*N* (%)	95% CI
IHD confirmed	104 (29.4)	24.6–34.1%
Heart conditions, other	27 (7.6)	4.8–10.4%
Observation, IHD not confirmed	129 (36.4)	31.4–41.4%
Discharged	39 (11.0)	7.8–14.3%
Non-heart conditions	50 (14.1)	10.5–17.7%
Lost to follow up	5 (1.4)	0.2–2.6%

**Table 4:** Non-heart conditions (*n* = 50).

	*N*	%
Pneumonia	15	30
Infection, others	6	12
Unspecific chest pains	6	12
Pain, others	5	10
Lipothymia	6	12
Epilepsy	3	6
Chronic Obstructive Pulmonary Disease	2	4
Dyspepsia	1	2
Kidney or urinary tract stones	1	2
Others	5	10

**Table 5:** Final diagnoses of the patients with prehospital diagnoses of Acute Myocardial Infarction (*n* = 48).

	*N* (%)	95% CI
Acute Myocardial Infarction	34 (70.8)	57.9–83.7%
Ischaemic Heart Disease, other	2 (4.2)	0–9.9%
Heart conditions, other	5 (10.4)	1.8–19.0%
Suspected AMI, IHD not confirmed	6 (12.5)	3.1–21.9%
Non-heart conditions	1 (2.1)	0–6.2%

## References

[1] Nielsen NK, Rasmussen S Heart statistics—focus on gender and social differences.

[2] Pope JH, Selker HP (2003). Diagnosis of acute cardiac ischemia. *Emergency Medicine Clinics of North America*.

[3] (1994). Indications for fibrinolytic therapy in suspected acute myocardial infarction: collaborative overview of early mortality and major morbidity results from all randomised trials of more than 1000 patientsFibrinolytic Therapy Trialists' (FTT) Collaborative Group. *The Lancet*.

[4] De Luca G, Suryapranata H, Ottervanger JP, Antman EM (2004). Time delay to treatment and mortality in primary angioplasty for acute myocardial infarction: every minute of delay counts. *Circulation*.

[5] Zeymer U, Arntz HR, Dirks B (2009). Reperfusion rate and inhospital mortality of patients with ST segment elevation myocardial infarction diagnosed already in the prehospital phase: results of the German Prehospital Myocardial Infarction Registry (PREMIR). *Resuscitation*.

[6] Feldman JA, Brinsfield K, Bernard S, White D, MacIejko T (2005). Real-time paramedic compared with blinded physician identification of ST-segment elevation myocardial infarction: results of an observational study. *American Journal of Emergency Medicine*.

[7] van ’t Hof AWJ, Rasoul S, van de Wetering H (2006). Feasibility and benefit of prehospital diagnosis, triage, and therapy by paramedics only in patients who are candidates for primary angioplasty for acute myocardial infarction. *American Heart Journal*.

[8] Holler CP, Wichmann S, Nielsen SL, Møller AM (2012). Large discrepancy between prehospital visitation to mobile emergency care unit and discharge diagnosis. *Danish Medical Journal*.

[9] Pope JH, Ruthazer R, Beshansky JR, Griffith JL, Selker HP (1998). Clinical features of emergency department patients presenting with symptoms suggestive of acute cardiac ischemia: a multicenter study. *Journal of Thrombosis and Thrombolysis*.

[10] Bruyninckx R, Aertgeerts B, Bruyninckx P, Buntinx F (2008). Signs and symptoms in diagnosing acute myocardial infarction and acute coronary syndrome: a diagnostic meta-analysis. *British Journal of General Practice*.

[11] Goodacre SW, Angelini K, Arnold J, Revill S, Morris F (2003). Clinical predictors of acute coronary syndromes in patients with undifferentiated chest pain. *Quarterly Journal of Medicine*.

[12] Body R, Carley S, Wibberley C, McDowell G, Ferguson J, Mackway-Jones K (2010). The value of symptoms and signs in the emergent diagnosis of acute coronary syndromes. *Resuscitation*.

[13] Arntz HR, Bossaert LL, Danchin N, Nikolaou NI (2010). European resuscitation council guidelines for resuscitation 2010 section 5. Initial management of acute coronary syndromes. *Resuscitation*.

[14] Breckwoldt J, Müller D, Overbeck M, Stern R, Schnitzer L, Arntz HR (2008). Prehospital care of acute coronary syndrome by anaesthetists. Prospective comparison with the care standards of cardiologists. *Anaesthesist*.

[15] Hemsey JKZ, Dracup K, Fleischmann K, Sommargren CE, Drew BJ (2012). Prehospital 12-lead ST-segment monitoring improves the early diagnosis of acute coronary syndrome. *Journal of Electrocardiology*.

[16] Lenfant C (2010). Chest pain of cardiac and noncardiac origin. *Metabolism*.

